# Changes in Cortical Activation Patterns in Language Areas following an Aerobic Exercise Intervention in Older Adults

**DOI:** 10.1155/2017/6340302

**Published:** 2017-03-06

**Authors:** Joe Nocera, Bruce Crosson, Kevin Mammino, Keith M. McGregor

**Affiliations:** ^1^VA Rehabilitation R&D Center for Visual and Neurocognitive Rehabilitation, Atlanta VAMC, Decatur, GA, USA; ^2^Department of Neurology, Emory University, Atlanta, GA, USA

## Abstract

Previous work has shown that older adults who evidence increased right inferior frontal gyrus (IFG) activity during language tasks show decreased sematic verbal fluency performance. The current study sought to evaluate if an aerobic exercise intervention can alter patterns of brain activity during a semantic verbal fluency task assessed by functional magnetic resonance imaging (fMRI). Thirty-two community-dwelling, sedentary older adults were enrolled to a 12-week aerobic “Spin” exercise group or a 12-week nonaerobic exercise control condition (Balance). Thirty participants completed their assigned intervention (16 Spin; 14 Balance) with pre- and postintervention assessments of a semantic verbal fluency task during fMRI and estimated VO_2_max testing. There was a significant increase in the change scores for estimated VO_2_max of the Spin group when compared to the Balance group. Semantic verbal fluency output within the scanner was also improved in the Spin group as compared to controls at postassessment. Group fMRI comparisons of IFG activity showed lower activity in the right IFG following the intervention in the aerobic Spin group when compared to the Balance group. Regression analysis of imaging data with change in both estimated VO_2_max and semantic verbal fluency was negatively correlated with activity in right IFG. The current work is registered as clinical trial with NCT01787292 and NCT02787655.

## 1. Introduction

Over the past few decades considerable attention has been devoted to examining the benefits of aerobic exercise on central nervous system plasticity. Aging research suggests that the positive effects of aerobic exercise involve higher order cognitive-executive processes, which are subserved largely by the frontal lobes [1–3]. Significantly, the frontal structures and related executive processes required for semantic verbal fluency are among the areas demonstrated to be most affected by aerobic exercise in humans [1, 4]. For example, Baker et al. (2010) reported that older participants with mild cognitive impairment who participated in an aerobic exercise regimen had improvements in semantic fluency, as assessed by the Delis-Kaplan Executive Function (DKEF) category test when compared to a contact controlled cognitive training group. Additionally, Voelcker-Rehage et al. [5] studied executive function in older adults engaging in a walking exercise program for one year. Participants in the aerobic exercise condition evidenced significant improvements in both category member generation and visual search acuity. In our own lab, a recent study demonstrated improvements in semantic verbal fluency in previously sedentary older adults following 12-week of aerobic, “Spin” cycling when compared to a control group [6]. A noted limitation in that study was our inability to identify the neural underpinnings promoting the semantic verbal fluency gains associated with increased cardiovascular fitness brought on by the aerobic exercise intervention. As such, we designed the current study to attempt to elucidate the neural mechanisms that may underlie improvements in semantic output associated with improved cardiovascular fitness in older adults.

Aerobic exercise has been increasingly associated with improvements in memory, executive function, and patterns of neural activity as assessed by fMRI [2–4, 7–11]. Recent fMRI evidence has also indicated that increased levels of aerobic capacity in older adults are also associated with improvements in language function and a more efficient neural recruitment array during a semantic verbal fluency task. For example, in a cross-sectional study, Zlatar et al., 2013, demonstrated that the neural recruitment array during a semantic verbal fluency task in physically active older adults resembled that of younger adults, while sedentary older adults showed decrements in suppression of areas that should be inhibited during the task. They went on to demonstrate that longer interhemispheric inhibition, as measured by transcranial stimulation, was associated with more negative task-related activity in the right and left posterior perisylvian cortex, suggesting that sedentary aging may result in losses in task facilitatory cortical motor inhibition [11]. As such, based on these findings, the losses of negative task-related activity may be mitigated by regular engagement in physical exercise [12, 13]. This indicates that older sedentary adults may be exhibiting a loss of inhibition associated with younger adults and physically active older adults (see also, [14]). However, as previous studies of exercise and language function have primarily been of a cross-sectional nature, we currently know little about how aerobic exercise interventions affect the neural substrates of semantic verbal fluency in previously sedentary older adults.

The aims of the present study were to test the effect of a 12-week aerobic exercise intervention against a nonaerobic control condition to investigate changes in semantic verbal fluency and its underlying neural activity in previously sedentary older adults. We hypothesize that increased aerobic capacity brought on by the aerobic exercise intervention will be associated with decreased blood oxygenation level dependent (BOLD) activity in right lateral frontal regions (Broca's homologue). Specifically, we hypothesize that the aerobic intervention would result in a decrease in recruitment of Broca's homologue which would correlate with behavioral improvement in a semantic verbal fluency language task.

## 2. Methods

### 2.1. Participants

In this 12-week randomized controlled trial, 32 participants were divided into an aerobic, Spin exercise group (Spin; *n* = 17) or a nonaerobic control group (Balance; *n* = 15) to equalize contact and monitoring. Study personnel explained the purpose, potential risks of the experiment and completed the informed consent process with each participant following protocols approved by the Emory University's Institutional Review Board (IRB) in compliance with the Helsinki Declaration.

Participants in this study were recruited from a volunteer database, which included elderly individuals (60 years and over). To meet inclusion criteria participants had to (1) be between 60 and 89 years of age, (2) report being sedentary, defined as not engaging in structured physical activity and/or not accumulating 30 minutes or more of moderate physical activity most days of the week, (3) have no history of depression, neurological disease, including Parkinson's disease, Alzheimer's disease, and multiple sclerosis or stroke, (4) report being right-handed, (5) report being a native English speaker, and (6) obtain physician's approval that it was safe for them to participate in the study. Exclusion criteria included (1) conditions that would contraindicate an MRI scan, (2) failure to provide informed consent, (3) hospitalization within the past 6 months, (4) inability to walk 400 meters, and (5) significant cognitive-executive impairment, defined as a score on the Montreal Cognitive Assessment (MoCA) of <24.

### 2.2. Aerobic “Spin” Intervention Protocol

Consistent with our previous study [6], the group exercise intervention began with 20 minutes of Spin aerobic exercise three times a week for 12 weeks on stationary exercise cycles and was led by a qualified instructor. Importantly, the time of each session progressed based on the recommendation of the instructor by 1-2 minutes as needed to a maximum time of 45 minutes per session. Exercise intensity began at low levels [50% of maximal heart rate reserve (HRR)] and increased by 5% every week (if deemed necessary by the instructor) to a maximum of 75% maximal HRR.

The Spin intervention took place in a climate controlled fitness facility. The instructor guided the participants through a 5-minute warm-up and then a work out phase that included steady up-tempo cadences, sprints, and climbs. During the workout phase the target HRR reserve was maintained by averaging increases and decreases in intensity/HR. The goal was to maintain a 10% offset from the HRR goal during the workout phase. Thus, participants were within target HRR on average across the session. All participants wore HR monitors (FT7 Polar® Heart Rate monitor) and were instructed each day of their target HR range. Staff members also monitored and tracked the HR to ensure adequate intensity throughout each session. Weekly meetings in which each participant's HR was reviewed served as a way to encourage those with low attendance or HR to improve their performance for the next week.

### 2.3. Control Intervention Protocol

Participants in the control group were equalized (frequency and duration) to the Spin group for contact and monitoring. As such they reported the same facility with the same interventionist; however, instead of progressive aerobic exercise they engaged in group balance, stretching, and light muscle toning exercises. Similar to the aerobic group, the Balance intervention began with an initial 20-minute session steadily progressing to 45 minutes over the course of the 12-week intervention. Heart rate was consistently monitored to assess general intensity during each session.

### 2.4. Assessments

All assessments were done no more than 10 days before the start of or 10 days after the conclusion of the 12-week intervention period. Assessment sessions did not exceed two hours to alleviate participant fatigue.

### 2.5. Cardiovascular Fitness Assessment

To validate that the Spin exercise was effective at increasing cardiovascular fitness when compared to control, participants performed a YMCA submaximal fitness test on a cycle ergometer. This submaximal test was used to estimate the participant's maximal oxygen uptake (VO_2_max) prior to and after the intervention period. The selected submaximal test is much better tolerated than a maximum exertion treadmill test in the selected population (sedentary older adults). The YMCA test uses an extrapolation method in which heart rate workload values are obtained at 2–4 points during stages of increasing resistance and extrapolated to predict workload at the estimated maximum heart rate (e.g., 220-age). Estimated VO_2_max is then calculated from the predicted maximum workload. Prior to beginning the test, the procedures were briefly explained and participants completed a 2-minute warm-up consisting of pedaling without load so that they could adapt to the ergometer for the first minute and then pedaling with a 0.5 kg·m load during the second minute. The YMCA submax test has an *R* = 0.86 with VO_2_max and a SEE = 10% of the predicted VO_2_max [15].

### 2.6. Cognitive Assessments

All participants completed a battery of neuropsychological tests to assess executive function and memory both before and after the interventions. The tests in the battery included the Controlled Oral Word Association (COWA) test (Letter and Semantic Fluency), the Hopkins Verbal Learning Test (HVLT), and forward and reverse digit span.

### 2.7. Scanning Protocol

During fMRI semantic verbal fluency acquisition, the participant's task was to overtly generate different exemplars of the respective category.

Similar to our previous work examining the neural underpinnings of semantic verbal fluency [14], a sparse temporal sampling approach was implemented to account for artifacts involved with overt speech. With a given repetition time (TR) (set at 5.83 seconds), image acquisition was delayed by 4 seconds during which participants were cued to make an overt response. Sagittal plane echo planar imaging was compressed into the final 1.83 seconds of each TR. Participants saw different categories (e.g., “flowers”) at the center of a 1024 × 768 pixel video screen while being in the scanner and would generate a word describing an object they associate with that category (e.g., “rose”). This consisted of 8 blocked semantic verbal fluency conditions, followed by a control condition (reading the word “rest” aloud) that afforded contrast between semantic engagement and motor speech production. All responses were recorded including errors of commission (semantically unrelated responses or repeats) and omission (no response). Control blocks were jittered from 3–5 TRs per block and were presented after each semantic verbal fluency block. Each functional run in the scanner included 3 blocks requiring naming 8 objects in 6 different categories, with each participant completing 3 of these runs for a possible total of naming 144 objects. A total of 74 images were acquired per run with the first two images designated as equilibration images to be discarded.

Error analysis on scanner response proceeded as follows. Correct responses consisted of a semantically related member of the provided category (e.g., “lion” for ANIMAL category). Two raters scored the responses and inconsistencies were resolved by interrater agreement. Incorrect responses were semantically unrelated utterances (e.g., “ball” for ANIMAL), filler words (e.g., “um, er”), or no response given. Failure to respond comprised 80% of errors in the test sample.

Functional images were obtained on a 3T Siemens Trio (Erlangen, Germany) platform with a whole-brain, 1-shot gradient EPI scan using a 12-channel RF receive coil with the following parameters: 240 × 240 mm FOV, 64 × 64 matrix (3.75 × 3.75 mm in-plane resolution), TR = 5830 ms, time of acquisition (TA) = 1830 ms, echo time (TE) = 25 ms, and flip angle (FA) = 70°. Image voxels were isotropic using a 3.75 mm slice thickness (no gap) with 32 slices acquired per image. A high-resolution T1-weighted 3D rapid acquisition gradient echo (MP-RAGE) scan (TE = 4.13 ms, TR = 2000 ms; FOV = 240 mm; FA = 8°; matrix size = 256 × 192 mm, 128 × 1.3 mm sagittal slices) was obtained to provide anatomic reference. A laser position system was used to align the participants within the bore of the magnet. Head motion was minimized using foam padding and careful instructions were given to the participant about avoiding motion.

### 2.8. Data Analysis: Behavioral Data

Statistical analyses were conducted using Microsoft Excel and JMP 12 (SAS Institute, Cary, NC). Potential group differences at baseline on demographic and psychometric parameters were evaluated using a between-subjects *t*-test. To evaluate pre-post-differences between groups, change scores for behavioral data were computed using the convention: change = pre − post. Intervention effects were examined by independent sample *t*-test on change scores to determine between-group differences for the variables of interest: cognitive battery, cardiovascular fitness assessment, and in-scanner semantic verbal fluency performance.

### 2.9. Data Analysis: Imaging Data

For fMRI image processing, Analysis of Functional NeuroImages (AFNI) software and FMRIB Software Library (FSL) were used. Images were skull-stripped using a BASH shell optimized version of FSL Brain Extraction Tool (optiBET) [16]. After removal of equilibration images (first 2 TR) and linear trend removal, echo planar images were aligned to the first image of the initial EPI run using FSL's nonlinear registration tool (fNIRT). To minimize the effect of motion due to speaking artifact, we used an independent components analysis (ICA) approach as implemented by FSL's MELODIC and FIX suites. After slice timing correction and application of a 5 mm FWHM Gaussian kernel blur to account for spatial differences between subjects, we performed MELODIC's component identification on every run for each individual participant. We then used FSL's standard trained classifiers as implemented in their FIX suite with a component inclusion threshold of 18 components to regress out noise parameters. The selection of 18 components was performed after evaluation of 12, 15, 18, and 20 inclusion components with 18 having the optimal sensitivity and specificity. Image transform matrices to 2 mm MNI-152 space were computed using FSL for both anatomic and echo planar imaging. After noise removal and image interpolation to standard space, we proceeded with a generalized linear model (GLM) regression approach evaluating semantic verbal fluency blocks against the control condition and baseline error term. AFNI's 3dDeconvolve program was used to calculate the GLM of activity against the control task (spoken word “rest”). A Block function was selected for the duration of the semantic verbal fluency block (8 TR) and regression coefficient beta weights were output for group analysis for each run.

To evaluate group differences as a result of the interventions, we performed a split-plot (2 between × 2 within) ANOVA as implemented in AFNI's 3dMVM [17] with intervention group as the between factor and timepoint (pre/post) as the within-subjects factor. 3dMVM tests allow groups with different *n* to be evaluated using a GLM. The specified generalized linear tests (GLT) afforded between and within group comparisons, as well as interaction effects while controlling for sphericity due to within-subjects comparisons. We used AFNI's 3dClustSim (compiled September 2015) program to correct for multiple comparisons with a voxel-wise threshold level *p* < .01 holding alpha at .01 for a minimum cluster size of 101 voxels at 2 × 2 × 2 mm^3^ (MNI space). We selected this conservative threshold in light of recent work discussing random field theory in cluster correction [18]. We additionally added False Discovery Rate curves as a threshold check on the multivariate modeling results using 3dFDR in AFNI. The corresponding False Discovery Rate at the selected voxel threshold yields a *q* ≤ .02 for all reported differences.

We additionally performed regression analyses using AFNI's 3dRegAna application to test for significant correlations between change scores in both semantic verbal fluency and change in estimated VO_2_max in prediction of BOLD activity in the postsession. As above, statistical thresholds were set to *p* < .01, alpha = .02 with a minimum cluster size of 101 voxels in MNI space using 3dClustSim.

## 3. Results

One participant from each intervention group did not return for follow-up testing so the final cohort included 30 older adults (16 in Spin; 14 in Balance; ***μ*** = 69.45 ± 6.12 years). The Spin and Balance group did not differ significantly at baseline in any characteristics (see Table 1).

### 3.1. Behavioral Data

**Figure 1:**
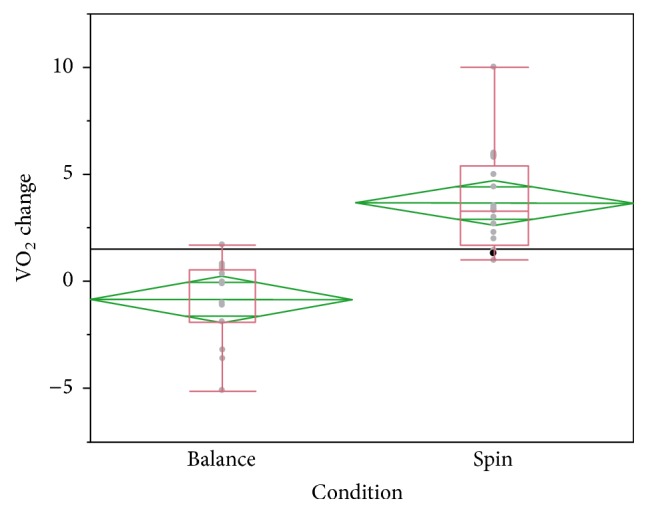
Difference in VO_2_ change after 12-week intervention in quantile plots. Means are presented as center green lines. Group differences in VO_2_ change are significant between the Spin and Balance groups (*p* < .01).

There was a significant difference between the change scores for the cardiovascular fitness assessment (estimated VO_2_max) of the Spin group (***μ*** = 3.85 ± 2.58) and the Balance group (***μ*** = −0.05 ± 1.05);* t*(29) = 4.63, *p* < .01 (Figure 1). Participants did not show significant changes in biometric assessments (weight, basal heart rate, and blood pressure) in either group after interventions.

Cognitive Test Battery: A trend was shown for better performance in the Spin group after intervention on both semantic verbal fluency outside the scanner (*t*(29) = 1.94, *p* = .06) and the Hopkins Verbal Learning Test (*t*(29) = 1.93, *p* = .06). Group differences in postintervention comparisons were not significant for tests of forward digit span and reverse digit span.

**Figure 2:**
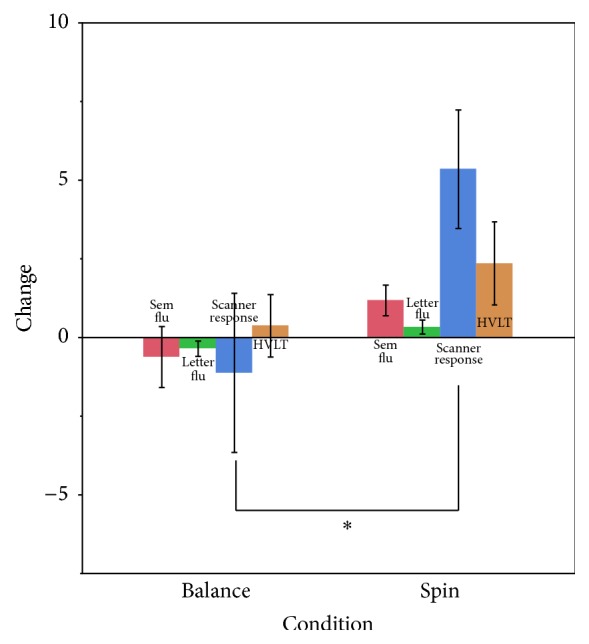
Group difference in the cognitive battery and in-scanner sematic fluency performance following the 12-week intervention. Sem Flu = semantic verbal fluency (outside scanner); letter flu = letter verbal fluency; scanner response = in-scanner semantic verbal fluency; HVLT = Hopkins Verbal Learning Test. *∗* denotes significant difference at *p* = .05.

In-scanner performance: in-scanner semantic verbal fluency performance improved after intervention in the Spin group as compared to the Balance control group (*t*(29) = 2.6, *p* = .01) (Figure 2). Error analysis between groups in postintervention assessment showed a significant change in error type. Errors of commission and omissions did not differ between groups in preintervention assessment (*t*(29) = 1.2, ns). However, during postintervention assessments, participants in the aerobic Spin training group showed fewer errors of omission (no response) than the Balance group (*t*(29) = 2.78, *p* < .01).

### 3.2. Imaging Data

We performed a between-subjects *t*-test on presession regression coefficients to test if there were differences at baseline between the two participant groups. Negligible group differences were evident at the selected threshold in this comparison on whole-brain analysis. No differences were evident in language eloquent cortices.

### 3.3. Postgroup Comparisons

**Figure 3:**
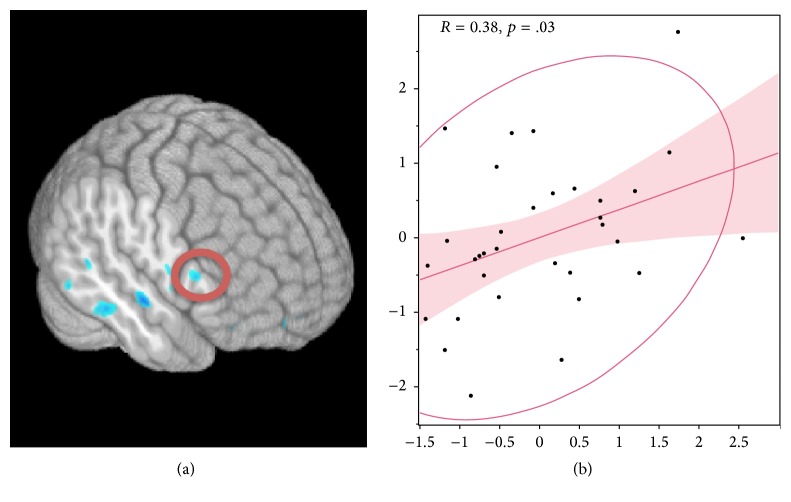
(a) presents a 3D whole-brain rendering of group differences after 3dMVM analysis of post session imaging data between Balance and aerobic Spin group. Color intensity (blue hue) denotes significantly lower levels of activity in aerobic Spin group correcting for multiple comparisons with a voxel-wise threshold level *p* < .01 holding alpha at .01 for a minimum cluster size of 101 voxels. (b) presents a correlation of VO_2_ change to change in inferior frontal activity after intervention. This indicates that the greater the VO_2_ change, the larger the change in right frontal activity. Ordinate axis is VO_2_ change and abscissa is change in frontal activity. All data is *z*-normalized.

Groups showed significant differences when comparing BOLD activity in postsession (see Table 2). Brain regions in the right hemisphere show significantly lower levels of BOLD activity in the Spin group. Importantly, decreased activity is shown in right hemisphere homologues of brain areas associated with semantic verbal fluency tasks including BA44/45, inferior temporal gyrus, and angular gyrus in the Spin group. Results of this analysis are shown in Figure 3 along with a correlation of change in VO_2_ with right inferior frontal activity during fMRI. As shown in the figure, change in VO_2_ was correlated with altered activity in right inferior frontal activity.

**Figure 4:**
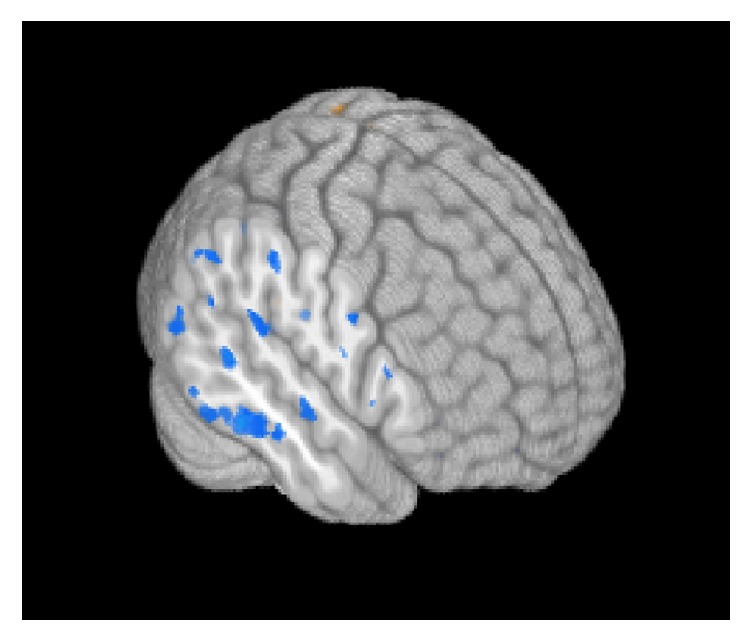
This figure presents a 3D whole-brain rendering of regression of VO_2_ change data with fMRI activity across all participants. Particularly in right hemisphere, the greater the VO_2_ change is in participants, the less likely they were to recruit right lateral frontal and right perisylvian language cortex. Orange color indicates increased fMRI activity with increased VO_2_ and blue indicates decreased fMRI activity with increased VO_2_. Data was corrected for multiple comparisons with a voxel-wise threshold level *p* < .01 holding alpha at 0.02.

Results from regression analysis on VO_2_ change data relating to postsession imaging results across participants are presented in Figure 4. As shown in the figure, increased VO_2_ at postsession was correlated with decreased activity in right inferior frontal activity (indicated in blue) but increased left lateralized activity (indicated in orange).

**Figure 5:**
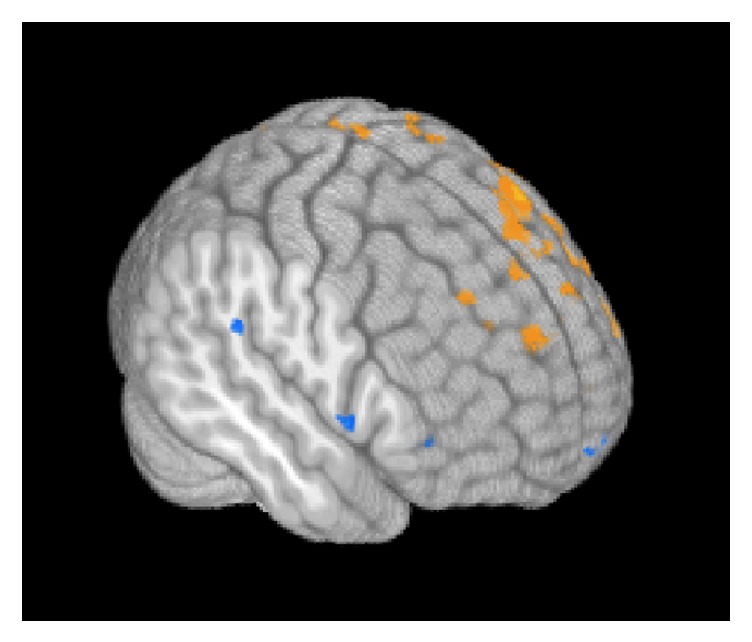
This figure presents a 3D whole-brain rendering of regression of in-scanner performance data with fMRI activity across all participants. A positive relationship was found between task performance and greater activity in left hemisphere (represented by orange). Activity in right language cortex was associated with decreased semantic fluency (represented in blue). Data was corrected for multiple comparisons with a voxel-wise threshold level *p* < .01 holding alpha at 0.02.

Results from regression analysis of postsession semantic fluency performance within the scanner with fMRI activity are presented in Figure 5. As shown in blue, there was a negative correlation between improved semantic fluency and right lateral frontal activity. This indicates that improved semantic fluency was associated with decreased reliance on right lateralized structures.

## 4. Discussion

The goal of the present study was to examine changes in brain activity during a semantic verbal fluency task in a previously sedentary cohort of older adults following 12 weeks of aerobic Spin exercise when compared to a nonaerobic, Balance control group. Consistent with our hypothesis, participants completing the aerobic Spin exercise condition improved their cardiovascular fitness level and showed improved semantic verbal fluency performance during a category member generation within the MR scanner as compared to the Balance control group. Additionally, when comparing group imaging data in the postsession controlling for variance between groups in the presession, the aerobic Spin group showed less positive BOLD activity in right lateral frontal, right superior temporal, and right angular gyrus.

As expected, the older adults exhibited positive BOLD activity in the right frontal operculum during the preintervention fMRI. These findings corroborate our previous findings indicating higher bilateral positive BOLD in older adults while performing an fMRI semantic verbal fluency paradigm. This increased bilateral activity is indicative of worse performance when compared to individuals who evidenced more left lateralized inferior frontal activity [14, 19]. In the present study, when comparing right lateral frontal activity and semantic verbal fluency performance across all participants, participants showed a strong negative correlation between positive BOLD activity and semantic verbal fluency output. That is, the more likely the individuals were to recruit right inferior frontal gyrus during semantic fluency, the worse their semantic verbal fluency output was. Importantly, the behavioral data are inline with and support the findings of a more efficient neural recruitment profile as measured by fMRI following the Spin intervention. To this point, the Spin group exhibited less BOLD activity after the intervention in right frontal regions while simultaneously demonstrating improvement in semantic verbal fluency output. Accordingly, the current findings suggest that an aerobic Spin intervention might facilitate a more efficient recruitment array during a semantic verbal fluency task. An intriguing finding in the current study is the difference in error types in semantic verbal fluency between groups evident after each intervention. Participants in the Spin aerobic exercise condition were less likely to make errors of omission after exercise than participants in the nonaerobic, Balance intervention. This may be associated with improved word finding within the semantic category selection task. Previous exercise interventions have also shown the efficacy of aerobic interventions in improving semantic verbal fluency [20, 21]. These findings support previous research indicating alterations in the neural recruitment profile, with a beneficial impact on executive performance following aerobic exercise [22, 23].

The finding of decreased activity in right inferior frontal regions being correlated with stronger semantic verbal fluency in older adults is, at face, seemingly at odds with a dominant model of hemispheric activity change respective of aging. The hemispheric asymmetry reduction in older adults (HAROLD) model has shown evidence that bilateral activity in older persons may be compensatory in nature [24]. This increased bilateral recruitment in older adults appears to be compensatory when considering generalized cognitive performance. However, we have shown that when task performance is compared with imaging data, increased bilateral recruitment tends to be detrimental to behavioral performance [10, 14]. This is consistent with findings from other laboratories that have reported increased error rates as associated with more bilateral recruitment in eloquent cortices [22, 25, 26]. However, as has been reported by numerous recent meta-analyses, it is extremely difficult to categorize bilateral BOLD activity as wholly compensatory or representative of inefficient processing [27, 28]. Among many numerous potential variables, in most aging-related imaging studies, physical activity is not included as a covariate. We interpret the current findings as potentially adding value to the debate on compensation or dedifferentiation in this respect. Much more work is needed to continue to explicate the complicated interrelationships of neural, vascular, and overall metabolic changes associated with aging that form the patterns of hemispheric activity changes so heavily modeled in the past few years.

The exact physiological mechanism responsible for the demonstrated changes in this exercise intervention study has yet to be determined. However, several candidates exist that need to be investigated to truly understand the mechanisms driving the changes evidenced in our exercise sample. Though beyond the scope of the present study, these mechanistic parameters may include, but are not limited to, an increase in brain-derived neurotrophic factor and other nerve growth factors [2, 8], changes in inhibitory systems function likely due to the neurotransmitter system gamma-aminobutyric acid ([29, 30]; see also [31]), and, most assuredly, increased vascular perfusion and optimized metabolic tone [32]. Strong evidence now exists showing that sedentary aging is associated with loss of cortical inhibition when compared with younger adults ([33–35] see [36] for recent review). Much of this literature has been informed by studies involving transcranial magnetic stimulation (TMS), but there is also growing evidence that cortical inhibition can be assessed using fMRI [30, 37] and that fMRI may be sensitive to aging-related changes in inhibition [14, 30, 37]. Aging-related changes in inhibitory function during language production, particularly in BA 44/45, have been reported with increasing frequency [19, 38, 39]. Future research should endeavor to incorporate multiple neuroimaging/neurophysiological techniques to better identify the physiological origin of the effect of exercise on verbal fluency.

An impressive finding of the current study is the relatively short amount of time (12 weeks) in which functional recruitment during a semantic verbal fluency task can be positively impacted by an exercise intervention. Baker et al. (2010) enrolled 33 participants with mild cognitive impairment over a 6-month span in which the participants engaged in 4 bouts of exercise per week. The participants followed a similar exertion schedule (though primarily treadmill-based) as the present study with heart rate targeted at 75–85% of HRR for the exercise sessions. After the 6-month training program semantic verbal fluency (measured by the DKEFS category fluency) significantly improved in females within the study cohort. Given this study was with patients diagnosed with mild cognitive impairment, the current findings may have clinical implications denoting the importance of beginning an aerobic exercise regimen prior to the onset of significant cognitive difficulties in older adults.

While Baker et al. (2010) did not use neuroimaging, numerous studies investigating the effects of aerobic exercise on executive functions have. In a seminal investigation, Colcombe et al. [40] demonstrated changes in activation patterns during a flanker task following 6-months of an exercise intervention. It should be noted, however, that the Colcombe study did not assess changes in the hemodynamic response at an earlier time point (e.g., 12 weeks); thus it is difficult to postulate when such changes might be observed. Additionally, the Colcombe study utilized a less intense (60–70% HRR) walking intervention, whereas we investigate a Spin exercise program designed to incorporate a higher intensity, interval-based workout (up to 75% of HRR with a 10% offset) within each session. Voelcker-Rehage et al. (2011) reported results on a 1-year walking intervention with previously sedentary older adults. This study (*n* = 44) reported improvements in executive function (visual search and flanker task) while showing reductions in recruitment of prefrontal regions during fMRI acquisition during related tasks. The group interpreted the reduced activation as an increase in processing efficiency within the prefrontal regions. However, their exercise program was relatively low intensity (~60% HR peak within age group) and involved 30–45 minutes of walking for 12 months. With inflexible exercise targets respective of duration and HR intensity, it is possible that participants may have acquired peak effects earlier in the intervention and then maintained a plateau with respect to their aerobic performance. As such the current intervention is modeled as a progressive and adaptive protocol that may offer consistent performance gains throughout the duration of the exercise program.

There are notable limitations to the current work that should be addressed. Most immediately, the sample size is somewhat low in the present report. While this is problematic from a data extensibility standpoint, it also is exciting given the fact that we were able to detect changes in a small number of individuals (i.e., high power). Given this, however, additional work is clearly warranted to attempt to better characterize neural activity changes as a result of aerobic interventions. Secondly, the present study cannot easily characterize the nature of how the BOLD signal changes. As such, it is difficult to differentiate vascular effects from the intervention as compared to neural changes. While it is impossible to completely dissociate one from the other using the present modality, we must acknowledge that improvements in vascular flow dynamics as a result of increased physical activity may better characterize the BOLD changes shown in the present study. Again, future work is needed with alternate methodologies to identify the focal mechanism of change presented here. Finally, as a methodological note, pre/postassessments using MRI introduce sensitivity/specificity variation due to differences in MR field characteristics. We utilized B0 field maps to assess changes between sessions in MR scanner function. While these did not reveal a gross overall change, physiological characteristics of repeated scans could not be accounted for. Statistically, we attempted to compensate for sensitivity differences by using a consistent and reasonable threshold for functional activity.

In conclusion, the present study shows that a 12-week aerobic exercise intervention (Spin) alters brain activity in language networks and may be associated with the improvement in sematic verbal fluency. Additional work is warranted to further evaluate the effects of aerobic exercise on the neural substrates of language production in aging.

## Figures and Tables

**Table 1:** Demographic and psychometric characteristics of participants at baseline.

	Spin group (*n* = 16, 10 females)	Balance group (*n* = 14, 6 females)
Age (years)	69.7 ± 6.34	72.09 ± 6.43
Education (years)	16.1 ± 2.77	15.46 ± 3.24
MOCA (maximum of 30)	28.21 ± 1.24	27.55 ± 1.12
Height (m)	1.68 ± 0.09	1.71 ± 0.11
Weight (kg)	93.15 ± 22.58	83.26 ± 12.34

MOCA: Montreal Cognitive Assessment; m: meters; kg: kilograms.

**Table 2:** Results from AFNI 3dMVM analysis of group differences (aerobic versus Balance) in fMRI activity during a semantic fluency task at postsession scan. Data was thresholded for multiple comparisons using a voxel-wise cluster size of 101 voxels, *p* < .02 corrected. Cluster size is in voxels.

Region	*X*	*Y*	*Z*	Cluster size
Right cerebellum	−2.5	57.5	−51.5	2063
Right inferior temporal gyrus	−57.5	32.5	−21.5	1972
Right angular gyrus	−40.5	51.5	26.5	1660
Right superior orbital gyrus	−23.5	−59.5	−0.5	1333
Right superior temporal gyrus	−55.5	8.5	−6.5	467
Right precuneus	−9.5	71.5	51.5	371
Left middle cingulate cortex	9.5	26.5	41.5	353
Right middle temporal gyrus	−57.5	46.5	2.5	281
Right inferior frontal gyrus (*P. triangularis*)	−47.5	−46.5	−1.5	581
